# Increase in short-term memory capacity induced by down-regulating individual theta frequency via transcranial alternating current stimulation

**DOI:** 10.3389/fnhum.2015.00257

**Published:** 2015-05-08

**Authors:** Johannes Vosskuhl, René J. Huster, Christoph S. Herrmann

**Affiliations:** ^1^Experimental Psychology Lab, Department of Psychology, Cluster for Excellence “Hearing4all”, European Medical School, Faculty for Medicine and Health Sciences, University of OldenburgOldenburg, Germany; ^2^Research Center Neurosensory Science, University of OldenburgOldenburg, Germany; ^3^Department of Psychology, University of OsloOslo, Norway; ^4^The Mind Research NetworkAlbuquerque, NM, USA

**Keywords:** short-term memory, working memory, theta-gamma coupling, tACS, phase-amplitude coupling

## Abstract

Working memory (WM) and short-term memory (STM) supposedly rely on the phase-amplitude coupling (PAC) of neural oscillations in the theta and gamma frequency ranges. The ratio between the individually dominant gamma and theta frequencies is believed to determine an individual’s memory capacity. The aim of this study was to establish a causal relationship between the gamma/theta ratio and WM/STM capacity by means of transcranial alternating current stimulation (tACS). To achieve this, tACS was delivered at a frequency below the individual theta frequency. Thereby the individual ratio of gamma to theta frequencies was changed, resulting in an increase of STM capacity. Healthy human participants (*N* = 33) were allocated to two groups, one receiving verum tACS, the other underwent a sham control protocol. The electroencephalogram (EEG) was measured before stimulation and analyzed with regard to the properties of PAC between theta and gamma frequencies to determine individual stimulation frequencies. After stimulation, EEG was recorded again in order to find after-effects of tACS in the oscillatory features of the EEG. Measures of STM and WM were obtained before, during and after stimulation. Frequency spectra and behavioral data were compared between groups and different measurement phases. The tACS- but not the sham stimulated group showed an increase in STM capacity during stimulation. WM was not affected in either groups. An increase in task-related theta amplitude after stimulation was observed only for the tACS group. These augmented theta amplitudes indicated that the manipulation of individual theta frequencies was successful and caused the increase in STM capacity.

## Introduction

Brain oscillations, in particular in the theta (3–8 Hz) and gamma (>30 Hz) frequency ranges, have been suggested to be key features in cognitive processes (Buzsáki, 2006; Lisman and Buzsáki, 2008; Lisman and Jensen, 2013), including memory performance (Düzel et al., 2010; Hanslmayr and Staudigl, 2014). We will focus on two subcomponents of memory, short-term memory (STM) and working memory (WM). Even though these two concepts are sometimes not clearly differentiated, we will use them to describe two theoretically different concepts: WM is a system that stores information, manipulates it for a short time and has limited capacity (Baddeley, 2012). STM in turn, does not involve a manipulation but rather refers to a mere storage of information (Conway et al., 2002; Engle, 2002; Baddeley, 2012).

**Figure 1:**
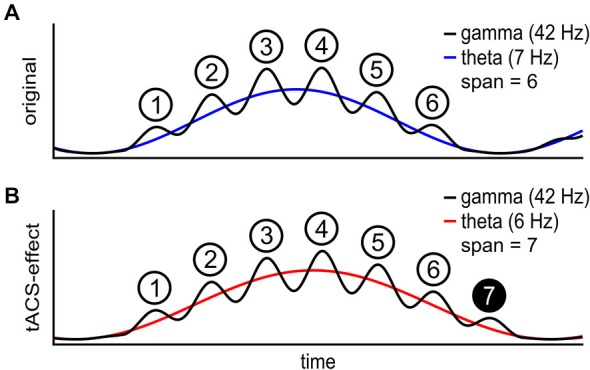
**Schematic of the theta gamma coding theory. (A)** The individual theta wave (blue) carries a gamma wave (black) with phase dependent amplitude. Each gamma cycle within one theta cycle represents one item in short-term memory (STM). The maximal number of items in STM is 6 in this case. **(B)** During transcranial alternating current stimulation (tACS) at a frequency below the individual theta frequency (red), STM capacity is increased to 7 items. At a lower theta frequency more gamma waves fit onto each theta wave.

Research over the past decades gathered evidence for the role of theta and gamma rhythms for WM and STM (Klimesch et al., 1996; Kahana et al., 2001). For example, an increase in amplitude of frontal midline theta activity was shown with higher load in WM tasks in both electroencephalogram (EEG; Gevins et al., 1997; Onton et al., 2005) and MEG studies (Jensen and Tesche, 2002; Osipova et al., 2006). It has also been shown that gamma band responses in EEG are stronger with memory items that are later remembered compared to those that are forgotten when memorizing was not instructed (Gruber et al., 2004). Similar findings are known for MEG (Herrmann et al., 2004; Jokisch and Jensen, 2007). Furthermore, the interplay between multiple frequencies has been shown to be of functional relevance for cognition (e.g., Axmacher et al., 2010; Jensen and Mazaheri, 2010; for comprehensive reviews see: Jensen and Colgin, 2007; Canolty and Knight, 2010). Several studies indicate that phase-amplitude coupling (PAC) between theta and gamma waves may serve as a mechanism to implement STM and WM in the human brain (Lisman and Jensen, 2013; Roux and Uhlhaas, 2014). The theta-gamma coding theory of STM and WM states that each gamma cycle represents one memory item while each theta-cycle represents the rehearsal of the list of all items held in storage (Lisman and Idiart, 1995; Jensen and Lisman, 1998; Lisman, 2010). This explains the limited capacity of these systems, which is determined by the number of gamma-cycles that “fit” onto one theta wave (Figure 1A). In support of this theory, it has been shown that the strength of PAC between theta and gamma frequencies as well as the underlying theta frequency depends on WM load (Axmacher et al., 2010). In a delayed match-to-sample task, increased PAC was found for remembered in relation to forgotten items (Köster et al., 2014). Most direct support for this theory was provided in an experiment showing that the theta to gamma cycle-length-ratio in fact correlates with STM capacity at fronto-central EEG electrodes (Kamiński et al., 2011).

Current evidence for an explanation of STM or WM capacity in terms of cross-frequency-coupling so far is correlative and therefore could be epiphenomenal. Therefore, in order to establish a causal relationship between the interplay of theta and gamma frequencies to STM or WM, we aimed at externally manipulating brain oscillations and thereby inducing changes in STM or WM characteristics. With a stable gamma frequency, down-regulating the ongoing theta frequency is intended to extend the STM capacity (Figure 1B).

Transcranial alternating current stimulation (tACS) is a relatively new method (Antal et al., 2008) with growing influence on research in brain oscillations (Antal and Paulus, 2013; Herrmann et al., 2013; Reato et al., 2013). tACS is a noninvasive method that modulates neural oscillations in a frequency-specific fashion by the application of alternating currents at the scalp. It thereby offers the possibility to test causal relationships between neural oscillations and brain functioning. It has been shown that tACS is in fact able to modulate brain oscillations and induce functional effects on both the behavioral and electrophysiological level. An increase of ongoing alpha amplitude was measured after 10 min of tACS at the individual alpha frequency (IAF; Zaehle et al., 2010) that can last at least 30 min after tACS offset (Neuling et al., 2013). Apart from this amplitude effect, modulations regarding a subject’s alpha peak frequency have also been reported (Helfrich et al., 2014; Cecere et al., 2015). In the first concurrent EEG-tACS study (Helfrich et al., 2014), stimulation at 10 Hz caused the variance of peak frequencies to decrease during tACS compared to a sham control group. This indicates a shift of the alpha peak frequencies towards the stimulation frequency in those subjects whose peak was not at 10 Hz before stimulation. The study also showed that directly after stimulation, the frequency effect disappears while the amplitude at the alpha peak frequency stays elevated. More behavioral evidence for a change in the endogenous alpha frequency stems from the analysis of timing of the sound induced double flash illusion (Shams et al., 2002). The timing window of the illusion was enlarged with tACS below the IAF and shortened with tACS above IAF (Cecere et al., 2015). In previous work, tACS proved capable to alter different cognitive functions for example memory functions in the theta (Polanía et al., 2012), auditory perception in the alpha (Neuling et al., 2012a), voluntary movement in the beta (Pogosyan et al., 2009) and bistable motion perception in the gamma range (Strüber et al., 2014).

Few studies used theta tACS to manipulate WM/STM performance so far. Reaction times in response to a delayed match-to-sample STM task were reduced by 6 Hz tACS that was phase-synchronous over parietal and frontal cortices, while desynchronized tACS had the opposite effect (Polanía et al., 2012). An increase in WM performance was found after tACS at an individual’s dominant theta frequency, as defined by the IAF minus 5 Hz, an effect observed in different WM tasks (Jaušovec and Jaušovec, 2014; Jaušovec et al., 2014). This was most prominent when tACS was delivered at left parietal areas. Effects of theta tACS on WM during stimulation were reported recently with prefrontal tACS at 4.5 Hz (Meiron and Lavidor, 2014) in a 2-back task.

**Figure 2:**
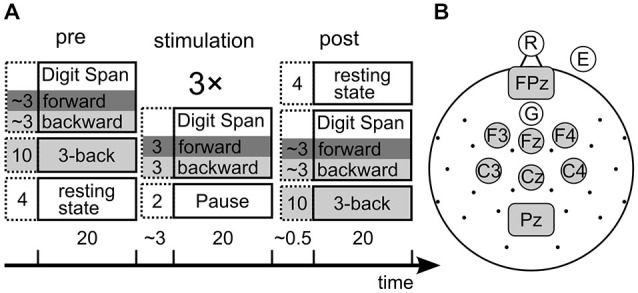
**(A)** Design of the experiment. Collumns show the three experimental phases, boxes show the tasks with their duration in minutes. The stimulation phase consited of three repetitions of the depicted tasks. Dark gray background marks STM tasks, light gray marks working memory (WM) tasks. The time bar indicates overall durations for each phase and pauses between phases. Note that the 3-back task was administered pre and post stimulation only, while the digit span tasks were assessed in all phases. **(B)** electroencephalogram (EEG) and tACS setup. Each dot or circle represent one measured electrode, central gray electrodes were pooled for EEG analysis. Gray rectangles represent rectangular stimulation electrodes.

One well established measure of STM is the forward digit span task (Wechsler, 2008) in which subjects are presented with lists of digits of increasing length that they need to remember for a short interval and then reproduce in either their chronological (forward) order. Because the forward digit span task does not involve any mental manipulation of the memory items whatsoever, it is a task that measures the mere storage capacity of STM (Engle, 2002). The same task with mentally reversing the list order is called backward digit span task and demands a mental manipulation (i.e., reversal) of the list. Therefore the backward digit span task can be considered a task that rather measures WM. As STM and WM do not seem to be two completely independent processes (Engle et al., 1999; Cowan, 2008; Aben et al., 2012), it is possible that both use the same storage system to memorize the presented items. To find effects of tACS on both WM and STM, forward and backward digit span performance is compared from before to after stimulation as well as in three blocks during stimulation (Figure 2A) for two groups of subjects: one receiving verum, and one a sham stimulation.

Previous research confirmed that fronto-parietal networks, which rely on theta oscillations as working language (Michels et al., 2010), are involved in both STM and WM processes (Olesen et al., 2004; Owen et al., 2005). Stimulation sites along the central line of the head have been shown to be successful in enhancing frequency-specific EEG amplitudes (Neuling et al., 2013; Helfrich et al., 2014) or manipulating endogenous frequencies (Helfrich et al., 2014; Cecere et al., 2015). Therefore stimulation sites for tACS were chosen to stimulate this wide-spread network in this experiment.

## Methods

### Participants

Thirty-five healthy subjects participated in this experiment after giving informed consent. Two were excluded from further analysis due to technical failures. Of the resulting 33 participants, all were right handed, 14 were female, and mean age was 25.74 ± 2.69. Participants were assigned to a tACS (verum, *N* = 17) and a sham (control, *N* = 16) group in a randomized single-blind fashion. Except for the actual stimulation, participants in both the tACS and sham group were treated the same to blind them with regard to the experimental groups. Groups did not differ in age (tACS: 25.72 ± 2.54, sham: 25.77 ± 2.93, *t*_(32)_ = −0.047, *p* = 0.963) or gender (tACS: 7 female, 10 male, sham: 7 female, 9 male, *χ*^2^(1) = 0.022, *p* = 0.881). The experimental protocol was approved by the ethics committee of the University of Oldenburg and was in accordance with the declaration of Helsinki.

### Experimental Tasks

We assessed performance in the forward and backward digit span task before, during and after three blocks of stimulation (Figure 2A). With the forward digit span task we tested the individual STM capacity, while backward digit span measured WM performance in this paradigm. Additionally the three-back task was measured before and after stimulation to have a second estimate of WM capacity. It was not measured during stimulation because the maximal length of tACS of about 20 min (Nitsche and Paulus, 2007) set time constraints on the design.

All tasks were controlled using Presentation (Version 14.08, Neurobehavioral Systems Inc, Albany, CA., USA). Responses were registered using a regular keyboard.

#### Digit Span Task

The task was designed in accordance with the WAIS-IV (Wechsler, 2008). Digits were shown one by one for 900 ms with a 600 ms inter stimulus interval. Forward digit span was assessed before backward digit span in all cases.

The stimulation phase consisted of three repetitions or blocks of the digit span task. During the stimulation phase, the task was changed to have a fixed duration of 3 min each direction in each of the three stimulation blocks. The list length was decreased or increased by one digit and task presentation resumed, depending on whether subjects reproduced three lists of a given length with or without errors, respectively. For example, if one subject reached list length six, and responds correctly to the first, second and third presentation of a list with six digits, the subsequently presented list will be increased to a total of seven digits. In case of three incorrect responses to the same list length, the subsequent list would contain five digits. As these two cases may alternate, it is possible and intended to repeatedly present the same subject with lists of the same length. As a consequence, this algorithms converges at a subject’s individual STM capacity limit.

Each of the three blocks started with a list two digits shorter than the maximal length achieved during the baseline assessment for both forward and backward trials respectively. Thereby an equal amount of backward and forward digit span data was assessed and the subject’s performance was kept around their individual limits.

As a response, the subject was instructed to enter the digits of the respective list via the keyboard and confirm their answer by pressing the space bar.

For statistical analyses, the mean list length of correctly answered lists was analyzed, as this constitutes a more reliable measure than the maximal correct list length (Wechsler, 2008), especially during the stimulation phase.

#### Three-Back Task

A randomized sequence of 200 letters was presented one by one on a computer screen for 1000 ms each with an interstimulus interval of 2000 ms (retention). Within the sequence, 40 targets were presented at random positions. A target was any letter that matched the letter presented three trials earlier. Responses were given only for targets and by pressing the space bar on a regular keyboard.

The measure of task performance we used was the net score (Haatveit et al., 2010), which is a subtraction of hit rate (“yes”-answers to targets) minus the false alarm rate (“yes”-answers to non-targets).

### EEG

EEG was recorded from 28 positions according to the 10–10 System plus right EOG (Figure 2B) using a BrainAmp amplifier (Brain Products, Munich, Germany), sintered Ag/AgCl electrodes and an elastic cap (Easycap, Falk Minow, Munich, Germany) inside an electrically shielded room. Recording reference was positioned on the tip of the nose and FCz served as ground. The EEG was digitized at a sampling rate of 1000 Hz and band-pass-filtered online between 0.1 and 250 Hz.

Signals were amplified to a range of ± 3.2768 mV at a resolution of 0.1 μV in order to be able to properly record gamma band activity. Note that EEG recorded during tACS at 1 mA produces an electric artifact in the range of several mV (Helfrich et al., 2014). Therefore, in our experiment most of the EEG channels during stimulation would have saturated and EEG could not have been restored from these data if it were recorded. With recording parameters during tACS on a lower resolution (e.g., 0.5 μV, range, ± 16.384 mV) gamma activity cannot reliably be measured, as gamma amplitudes are usually below 0.5 μV (e.g., Naue et al., 2011).

### Electrical Stimulation

Electrical stimulation was applied using a battery-operated stimulator system (Eldith, Neuroconn, Ilmenau, Germany). The stimulator emitted a sinusoidal alternating current to the participant’s scalp via two sponge-electrodes (5 × 7 cm, Neuroconn, Ilmenau, Germany). The electrodes were fixed at positions FCz and Pz (Figure 2B) underneath the elastic EEG cap with impedances below 10 kΩ.

Stimulation strength was set individually below the participant’s phosphene or perception threshold (min/max/mean/std: 400/1300/843/260 μA) with no difference between tACS and sham control group (*t*_(31)_ = −1.095, *p* = 0.282, two-tailed). Stimulation strength was determined for each subject by administering a staircase procedure, starting at 100 μA. After each upward step of 100 μA, the subject was asked to report any sensations related to tACS. Once the subject reported either phosphenes, skin sensations or the like, the final stimulation strength was adjusted 100 μA below that threshold. Current was build up and reduced for 10 s before and after stimulation (tACS) or without stimulation in between (sham). Over all, subjects in the tACS group received ca. 18 min of tACS over the three blocks.

**Figure 3:**
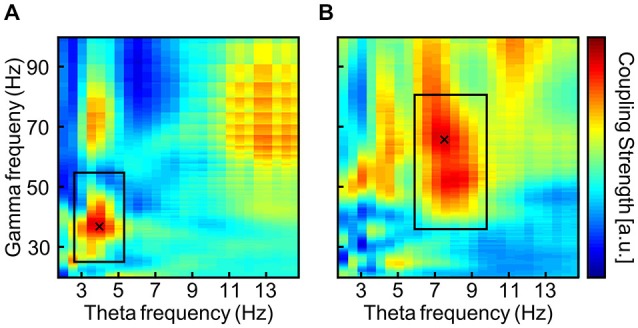
**Typical output of the phase-amplitude coupling (PAC) analysis via envelope-to-signal correlation (Onslow et al., 2011) from two subjects (A,B)**. The rectangles are drawn to surround a local maximum in the theta and gamma band. Thereby PAC maxima in other frequency bands that smear into the theta and gamma bands can be avoided. PAC values are normalized to each subject’s maximal PAC strength and are displayed color coded with warm colors indicating stronger PAC. The cross marks the maximum within the frequency area of interest.

An *ad hoc* analysis of task-related EEG during the 3-back task from the pre-stimulation phase was administered to define the individual theta frequency by means of PAC (Bruns and Eckhorn, 2004; Onslow et al., 2011). See Figure 3 for a typical outcome of two subjects. A frequency area of interest was manually adjusted around the local maximum within the margins of theta and gamma range in order to avoid maxima at the borders of the theta and gamma range (Black rectangles in Figure 3). The theta frequency that showed maximal coupling to any gamma frequency within the specified frequency range of interest was reduced by tACS such that STM capacity should increase by one item. As an example, one subject may show strongest theta-gamma PAC for 7 and 42 Hz, which would theoretically result in a STM capacity of 42/7 = 6 items (Figure 1). In the first block of tACS, the stimulation frequency would be adapted such that, 42/theta = 7 items, resulting in a 6 Hz stimulation. In the following blocks, the stimulation frequency was adjusted according to the same criterion increasing the STM capacity even further.

Mean stimulation frequencies used for tACS/sham group were 4.62/4.78 (std: 1.38/1.28), 4.12/4.27 (std: 1.16/1.08), 3.73/3.87 (std: 1.00/0.94) in the three stimulation blocks, respectively.

### EEG Data Analysis

All data analyses were performed using MATLAB (Version 7.11.0, The Mathworks Inc, Natick, MA, USA) and functions of the EEGLAB toolbox (Delorme and Makeig, 2004).

Task-related 3-back EEG was split into epochs of 2 s starting at the offset of a letter presentation to cover the retention phase between two letters. Epochs containing high frequency artifacts (mainly produced by muscle activity) were automatically detected and removed. The remaining epochs were then low-pass filtered at 80 Hz and resampled to 250 Hz. Eye-blink artifacts were rejected using an automated individual method to find extreme values (Zaehle et al., 2011). Remaining epochs from 6 fronto-medial channels (Figure 2B) were then analyzed for PAC in the *ad hoc* analysis.

Task-related EEG from the digit span task pre- and post-stimulation was low-pass filtered with an infinite impulse response filter at 80 Hz, and down sampled to 250 Hz *post hoc*. Independent component analysis (ICA) was used to remove ocular artifacts (Jung et al., 2000a,b). The backprojected EEG was then epoched from −600 to 900 ms around digit onset resulting in epochs of 1.5 s Fast Fourier Transformations were calculated and averaged over all epochs and the 6 fronto-medial channels leading to a frequency resolution of 0.66 Hz. Frequency spectra were pooled over forward and backward digit span tasks.

## Results

### Behavioral

**Figure 4:**
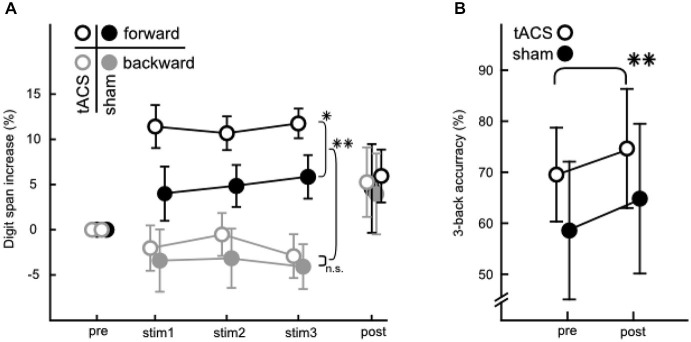
**Behavioral data from the WM and STM tasks. (A)** Percentaged increase of correctly answered lists relative to pre-stimulation in the digit span task. Digit spans were assessed pre-, during (stim 1/2/3) and post-stimulation. Forward (black) and backward (gray) digit span performance are depicted for tACS (open circles) and sham (filled circles). Note the significant differences between groups in the forward digit span task during stimulation and between forward and backward condition. **(B)** Performance in the 3-back task before and after the stimulation phase for tACS (open circles) and sham stimulated groups (filled circles). Values are net scores (hit rate—false alarm rate). Error bars in both A and B denote ± one standard error of the mean. Significance levels are indicated by asterisks (*p < 0.05, **p < 0.01), all other comparisons were not significant.

A descriptive overview over behavioral performance measures can be found in Figure 4 for the digit span (A) and 3-back (B) task. Figure 4A depicts increases in digit span performance relative to pre-stimulation.

#### Digit Span Task

An ANOVA (3 × 2 × 2) with factors stimulation *block* (stim1/2/3), *direction* (forward/backward) and *group* (tACS/sham) on mean list lengths from the digit span task recorded during stimulation (stim 1/2/3, Figure 4A) revealed that performance levels in backward and forward digit span differed significantly (main effect of *direction*: *F*_(1,31)_ = 67.111, *p* < 0.001, *η*^2^ = 0.684). This effect was to be expected since performance is known to be lower in the backward than the forward digit span task (Wechsler, 1945; Rosen and Engle, 1997). More importantly, the analysis also indicated that the aforementioned differences between the forward and backward digit span tasks varied between groups (interaction between *direction* and *group*: *F*_(1,31)_ = 6.832, *p* = 0.014, *η*^2^ = 0.181). Specifically, the forward digit span performance was significantly better in the tACS than the sham control group (planned comparison, *F*_(1,31)_ = 4.962, *p* = 0.033), whereas no such effect was found for the backward condition (*F*_(1,31)_ = 0.018, *p* = 0.894). Thus, tACS below the individual theta frequency specifically modulated performance during the forward digit span, but not during the backward digit span task. No general increase in performance was observed over the course of the three stimulation blocks.

An increase of digit span performance from before to after stimulation was tested using a separate ANOVA. These data were not analyzed together with data from during stimulation, because they were assessed using different experimental procedures. The pre- and post-stimulation assessments were conducted in accordance with the WAIS-IV (Wechsler, 2008). The measurement was aborted after the individual limit was reached. During stimulation the task was designed to measure an increase in performance and so it was adapted such that most trials presented dealt with list lengths around the individual capacity in a fixed time.

The analysis of digit span performance pre- and post-stimulation using a 2 × 2 × 2 ANOVA with factors *measurement* (pre/post), *direction* (forward/backward) and *group* (tACS/sham) on mean list lengths indicated that performance levels differed between forward and backward digit span. This effect was present at both, the baseline measurement before and the control measurement after stimulation (main effect of *direction*: *F*_(1,31)_ = 33.328, *p* < 0.001, *η*^2^ = 0.518). Performance within the two groups was similar before and after stimulation. Thus, tACS did not alter task performance differently between groups beyond the stimulation period. A marginally significant effect of *measurement* (*F*_(1,31)_ = 3.864, *p* = 0.058, *η*^2^ = 0.111) was detected that might be the result of continued task practice.

#### 3-Back Task

Further, a 3-back task was administered to assess the persistence of WM modulations from before to after stimulation (Figure 4B). Net scores (hit rate—false alarm rate) (Haatveit et al., 2010) were analyzed with a 2 × 2 ANOVA with factors *measurement* (pre/post), and *group* (tACS/sham). Neither the main effect of *group* nor the interaction of *group* with *measurement* were significant. Hence, no effect of tACS could be found when analyzing the 3-back performance. A significant change of performance from before to after stimulation (main effect of *measurement*: *F*_(1,31)_ = 9.835, *p* = 0.004, *η*^2^ = 0.241) could be demonstrated, however, which did not differ between groups, indicating that subjects in both groups similarly improved performance with increased familiarity with the task. Note that the 3-back task was not measured during tACS, and thus this analysis does not rule out a potential effect of tACS on task performance when measured online to tACS administration.

### EEG

**Figure 5:**
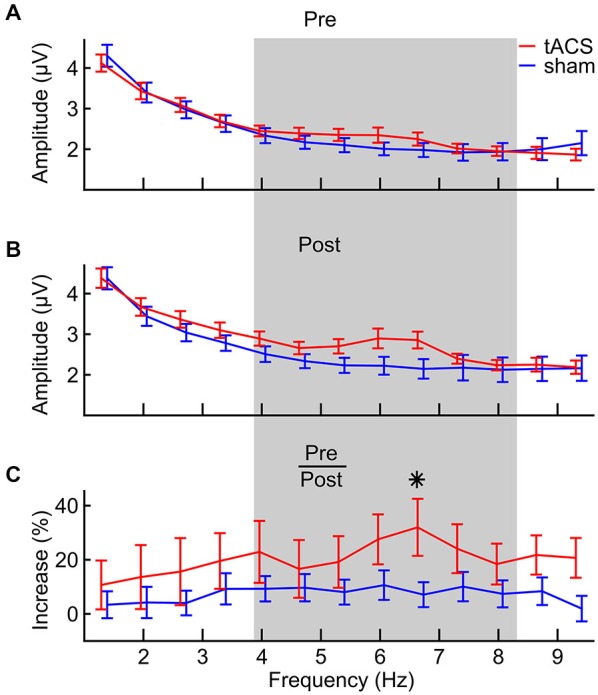
**FFT spectra of task-related EEG from the digit span task (A) pre-stimulation, (B) post-stimulation and (C) the relative increase from pre- to post-stimulation for both tACS (red) and sham (blue) groups**. The gray area defines the frequency area of interest between 4 and 8 Hz that was statistically analyzed. Error bars denote ± one standard error of the mean. The asterisk indicates a significant (*p* < 0.05) difference between groups.

In Figure 5, the frequency spectra from the digit span task before (A) and after (B) stimulation are depicted, together with frequency specific amplitude increases in percent relative to pre-stimulation (C). Spectra were calculated on epochs around the onset of each digit in each correctly answered list, pooled over forward and backward digit span and over 6 fronto-central electrodes (Figure 2B). At 6.7 Hz, a prominent difference can be seen between groups. Statistical analysis was done on the relative amplitude changes (Figure 5C) and for frequencies between 4 and 8 Hz (Figure 5C, gray shading). More specifically, EEG theta amplitudes from the digit span task after stimulation were analyzed relative to amplitudes from the baseline digit span assessment before stimulation in a 7 (*frequencies*) × 2 (*groups*) ANOVA. This analysis revealed that amplitudes increased differently over frequencies (main effect of frequency: *F*_(1,6)_ = 2.312, *p* = 0.035, *η*^2^ = 0.069) and that this effect is modulated by tACS (interaction of *frequency* and *group*: *F*_(1,6)_ = 2.404, *p* = 0.029, *η*^2^ = 0.072). Thus, amplitude increases were shown to be different between the two groups, with certain frequencies showing a stronger difference than others. A significant planned comparison at the peak theta frequency after stimulation (6.7 Hz) revealed that the *group* times *frequency* interaction is driven by frequencies in the medial theta range (*F*_(1,31)_ = 4.476, *p* = 0.042).

## Discussion

In this study, we demonstrated that tACS can enhance STM performance for the time of stimulation. The capacity limit of STM was augmented as a result of tACS stimulation and as predicted by the theta-gamma coding theory (Lisman and Idiart, 1995; Jensen and Lisman, 1998; Lisman, 2010). The EEG results of this study suggest that theta tACS successfully modulated individual theta oscillations. Thus, tACS proved to be a powerful tool for the investigation of causal relationships between neural oscillations and behavior.

Taking the conceptual differentiation between the two versions of the digit span task into account, the results of this study can be fully explained by the theta-gamma coding model (Lisman, 2010). Looking at the different performance levels in forward in contrast to backward digit span, it is obvious that less items can be stored in the backward condition than in the forward condition (Rosen and Engle, 1997). If performances in both the backward and forward digit span tasks depend on the same structures to store information, then the storage capacity of STM is not fully used in the backward digit span task. An extension of this storage would have no effect on the performance regarding the backward digit span task. This in turn means that a performance limit in the backward digit span task is caused by some other process different from the mere storage of items. One likely process to cause this limitation is the additional manipulation of items, sometimes referred to as executive attention (Engle et al., 1999).

In our study, like in previous studies, the amplitude of a stimulated frequency stays elevated after tACS offset (Zaehle et al., 2010; Neuling et al., 2013; Helfrich et al., 2014). We administered tACS below the individual theta frequency, and therefore a shift in theta frequencies should be expected. Behavioral results indicate that the effect of tACS quickly vanishes after stimulation offset. We therefore believe that the induced shift in individual peak frequencies due to tACS also disappears immediately after tACS offset since otherwise an elevated STM performance should have been observed after stimulation as well. Unfortunately, in the current study, we were not able to analyse the EEG measured concurrently with tACS due to the strong tACS artifact which can only be removed if the tACS intervals are an integer multiple of the EEG sampling rate. Due to our inter-individual variation in tACS frequency this was not given. Therefore, the presence of a frequency shift during stimulation cannot be directly demonstrated in this study. Notwithstanding, we found an amplitude increase in the theta band after stimulation which we interpret as indirect evidence for a successful frequency modulation. In the first study that analyzed EEG recorded during tACS and with recording parameters that prevented saturation and fulfilled the afore mentioned criterion for stimulation frequencies, 10 Hz tACS has led to an increase in alpha amplitude and a decrease in variance of alpha peak frequencies, i.e., a frequency shift (Helfrich et al., 2014) during stimulation. After stimulation offset, however, the variance of alpha peak frequencies changed back to the pre-stimulation status, but the mean amplitude peak was still elevated (Helfrich et al., 2014). Vossen et al. (2014) recently showed that tACS slightly below the IAF can elevate the amplitude at IAF after stimulation offset. In their article they suggest a model based on spike timing dependent plasticity to explain that effect. These findings lead us to the interpretation that tACS in our study in fact changed individual theta frequencies and STM performance during stimulation. However, only the enhancement of theta amplitudes remained visible after stimulation while the shifted frequency returned to its pre-stimulation value immediately after the end of stimulation.

Previous studies manipulating WM or STM with theta tACS were heterogeneous in their outcome. Different results have been reported depending on task and electrode position. Also, most of the previously published results find behavioral aftereffects of theta tACS in contrast to our study. A post-tACS effect could be found in the visual array comparison task (Jaušovec and Jaušovec, 2014) and backward digit span (Jaušovec et al., 2014) with tACS at left parietal regions. Stimulation of the right parietal cortex led to aftereffects in forward digit span and in the 1 and 2-back task while left frontal stimulation only affected 1-back performance (Jaušovec et al., 2014). Bilateral frontal tACS had an effect on a 2-stream 2-back task during stimulation, but no offline effect in a corresponding task (Meiron and Lavidor, 2014). The multitude of stimulation protocols and tasks used makes a general interpretation of previous and current findings difficult though, but may well explain heterogeneous outcomes.

Earlier studies showed substantial diversity of tasks used that could in part explain the varying outcomes. Some researchers used spatial versions of STM tasks (Jaušovec and Jaušovec, 2014; Jaušovec et al., 2014), or combined this with an auditory letter n-back task with n between 1 and 3 (Jaušovec et al., 2014). There still is a discussion on the presence of different WM storage systems for auditory and visual items, partially fostered by the observation that visual capacity is usually found to exceed auditory capacity (Saults and Cowan, 2007; Fougnie and Marois, 2011). It is therefore problematic to compare WM performance over modalities. Also, verbal and spatial information may be stored in separate brain structures (Baddeley, 2000).

Yet, the diversity of findings between the cited studies may not be explained by the variety of tasks alone. It may also be explained by differences in stimulation protocols in at least two ways. In contrast to the previous studies, we first determined the individual theta frequency and stimulated relative to that, not relying on a frequency that was kept constant across subjects (Meiron and Lavidor, 2014). We measured the theta frequency directly by maximal theta-gamma PAC during task performance instead of estimating it on the basis of the individual alpha peak frequency at resting state (IAF—5 Hz) (Jaušovec and Jaušovec, 2014; Jaušovec et al., 2014). The current method seems more accurate for our purpose, since we individually search for the theta frequency that is most strongly coupled to a gamma frequency. Another advantage of our procedure is that we estimate the individual theta frequency from EEG recorded during task performance and not during resting state. The stimulation frequencies in our study were also descriptively lower (mean: 4.62, 4.12, 3.73 Hz) than those used in other studies for example 5 Hz (Jaušovec and Jaušovec, 2014; Jaušovec et al., 2014) and 4.5 Hz (Meiron and Lavidor, 2014) since our goal was to decrease the individual theta frequency and therefore stimulate below the estimated individually dominant theta frequency.

At last, the stimulation positions may as well explain differences between studies to some extent. With our electrode positions, we intended to stimulate a wide network of parieto-frontal areas in order to modulate inter-areal communication. Another approach would be to aim at a certain part of the parietal (Jaušovec and Jaušovec, 2014; Jaušovec et al., 2014) or frontal cortex (Meiron and Lavidor, 2014). With different electrode positions, certainly different brain areas are stimulated even though tACS does not focally stimulate the underlying tissue (Neuling et al., 2012b) and therefore different behavioral outcomes are to be expected.

In this experiment we can only provide indirect evidence for a manipulation of theta frequencies by showing the effect of increased theta amplitudes after stimulation. Consequently, further research is needed measuring and analyzing EEG concurrently to tACS. In an alternative scenario, however, our stimulation protocol may have only enhanced theta amplitudes but not changed its frequency. By this enhancement of theta amplitudes, STM capacity could have been modified only indirectly as a side effect of enhanced attention (Gevins et al., 1997; Jensen and Tesche, 2002). We strongly argue for our line of argumentation for two reasons: First we find enhanced theta amplitudes after tACS offset without a corresponding behavioral effect. If an increase in theta amplitude is responsible for the observed effects, STM performance should stay elevated after stimulation. Second, converging evidence suggests that tACS can change an endogenous frequency during its application in behaving human subjects (Helfrich et al., 2014; Cecere et al., 2015) and *in vitro* (Ozen et al., 2010; Schmidt et al., 2014).

Only when directly observing the frequency shift while tACS was turned on Helfrich et al. (2014) the alternative explanation that our stimulation protocol only enhanced theta amplitudes can be ruled out.

A significant change of STM performance by theta tACS has been reported in an experiment modifying the phase of parietofrontal theta oscillations (Polanía et al., 2012). In this study, tACS was applied between P3 and Fz, resulting in 180° phase lag between the two positions (desynchronization) in another condition, P3 and F3 were stimulated in phase by using the central electrode as a return electrode (synchronization). The authors report an increase of reaction times to a delayed match to sample task in response to the desynchronized tACS, and a reverse effect for phase-synchronized stimulation. Our finding seems to contradict these reports since our electrode setup resembles the desynchronized stimulation. Although both results are discussed as a change in STM performance, these findings are very different in nature. A delayed match to sample task does not measure STM capacity, thus these results suggest a different interpretation. The application of desynchronized theta oscillations to left parietal and frontal cortices lead to a slower processing, which does not necessarily translate to reduced STM capacity. In conclusion, our results do not contradict Polanía et al. (2012), they only measure different features of STM, i.e., STM capacity instead of processing speed. In addition, the interpretation of reaction times does usually not make sense in a digit span task since subjects are deliberately given the opportunity to reconsider their response and are instructed to respond as correct, as possible.

In contrast to the previously discussed publications, our aim was to externally modulate the individually dominant theta frequency in order to induce an increase in STM capacity. This hypothesis was derived from the theta-gamma coding theory of STM/WM and directly tests this theory. All previously mentioned studies did not explicitly try to change a frequency but rather modulate theta amplitudes. None of the studies showed a change in the oscillatory response of the brain as manipulated by tACS by analyzing the frequency composition of EEG recorded after or during stimulation.

## Conclusions

Our study showed that tACS below the individual theta frequency can be used to augment the individual capacity of STM as measured via the forward digit span task. WM performance in the backward digit span task and the 3-back task is not altered by this treatment, neither during nor after stimulation. As a result of theta tACS, the amplitude of theta frequencies stays elevated beyond stimulation. This outcome adds causal evidence to the theta-gamma coding theory of STM (Lisman and Idiart, 1995) and thereby backs up previous correlational results that supported the theory. This study deepens the knowledge about both STM and the method of tACS. This knowledge can be used first to gain a better understanding of diseases causing symptoms affecting STM performance as well as to eventually develop a tACS based treatment thereof. It also suggests that tACS can in fact modulate the frequency of EEG theta oscillations and thereby provide a useful method to study other frequency specific cognitive functions.

## Author Contributions

Designed research: JV, RJH, CSH; Performed research: JV, RJH; Contributed to analytic tools: JV, RJH, Analyzed data: JV, RJH; Wrote the paper: JV, RJH, CSH.

## Conflict of Interest Statement

The authors declare that the research was conducted in the absence of any commercial or financial relationships that could be construed as a potential conflict of interest.
